# Semi-Destructive and Non-Destructive Tests of Timber Structure of Various Moisture Contents

**DOI:** 10.3390/ma14010096

**Published:** 2020-12-28

**Authors:** Justyna Jaskowska-Lemańska, Elżbieta Przesmycka

**Affiliations:** 1 Department of Geomechanics, Civil Engineering and Geotechnics, AGH University of Science and Technology, al. Mickiewicza 30, 30-059 Kraków, Poland; 2 Faculty of Architecture and Fine Arts, Andrzej Frycz, Modrzewski Krakow University, ul. Herlinga-Grudzińskiego 1, 30-705 Kraków, Poland; e.przesmycka@365-afm.edu.pl

**Keywords:** timber structures, non-destructive tests, semi-destructive tests, X-ray computed tomography, wood moisture, natural hazards

## Abstract

The condition of heritage and historic timber constructions depends on how they are exploited. Numerous environmental factors degrade the physical and mechanical properties of timber and hence, affect the load-bearing capacity of constructions. As a result, frequent evaluations of their technical condition become necessary. Currently, modern technologies allow for extensive diagnostics of timber constructions using non-destructive and semi-destructive methods; yet, in contrast to classical laboratory tests, there is insufficient knowledge of the impact of individual factors on the results of such studies. This article presents an assessment of the influence of the moisture content of timber elements on the results of ultrasonic stress wave, sclerometric, and resistance drilling tests. Additionally, computed tomography scans were performed on selected samples to demonstrate the destruction mechanism occurring during the semi-destructive tests. The research involved three types of wood: pine, spruce, and fir of different moisture contents. The results reveal a strong relation between the moisture of timber and all the tests conducted in terms of both hygroscopic and capillary moisture.

## 1. Introduction

Wood, as a construction material, exhibits high durability in favourable conditions, but, under typical conditions of building operation, exposed to a number of disadvantageous factors, it deteriorates faster than other building materials. Such properties of timber constructions require an assessment of their technical condition. The problem of load-bearing capacity assessment of timber construction members occurs on occasions of renovation or modernization of historic and heritage objects [1,2,3,4].

Historical wooden structures concern all types of buildings or structures entirely or partly wooden, which have cultural significance. Their preservation and protection are a necessary and unquestionable action. In Poland, more than 10% of historic objects are wooden and were built in the timber framing method; in addition, most of the remaining buildings have horizontal building envelopes with timber construction. However, due to challenges related to the proper assessment of technical condition, on many occasions, timber objects or elements, both in Poland and worldwide, are being replaced completely. The consequence of this is a gradual disappearance of historical timber structures and, consequently, impoverishment of cultural heritage [5]. In recent years, several documents have been created recommending particular types of research aimed at estimating the parameters of wood in structures [6,7]; unfortunately, the interpretations of the majority of them do not take into account the factors to which wooden objects may be exposed.

**Figure 1:**
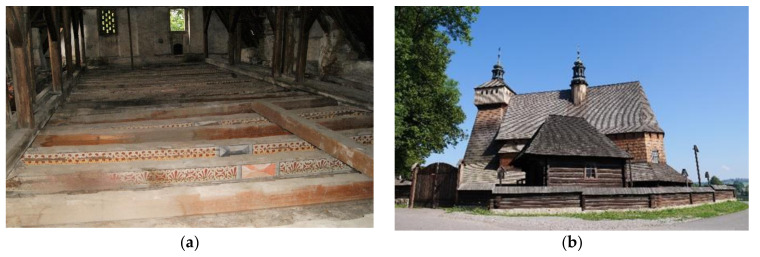
(**a**) The beam ceiling of the Gorzanów Palace—high humidity of the elements resulted from long-term leakage in the roofing; (**b**) The church in Haczów—repeatedly flooded by floods.

The most dangerous threat to the durability of timber structures is humidity. Both objects and structural elements are exposed to weather conditions and other accidental factors, which significantly affect the results of their testing. Knowledge on the influence of moisture on the results of the examination of wooden structures may enable the maintenance of more than their quantity. Figure 1 shows examples of historical timber elements and objects that were exposed to water at different intensity levels.

Tests of construction elements can be divided, as far as their impact on the structure is concerned, into destructive (DT), non-destructive (NDT), and semi-destructive tests (SDT). Destructive tests require taking numerous samples from the construction, which is usually unacceptable in the case of historic and heritage buildings. Thus, norms and standards recommend, first of all, the use of NDT and SDT methods [6,8,9].

Among non-destructive methods, which do not leave any permanent traces, the following subgroups can be found: visual strength-grading [10], weighting, and methods based on the propagation of electromagnetic waves including ultrasound methods [11,12], as well as advanced X-ray and gamma-ray tests [13,14,15]. The second subgroup of tests is characterized by a low impact on the object. During such studies, only small holes are made in the specimen, hence the name semi-destructive methods. In this subgroup, a few classes of methods are distinguished depending on the degree of impact:

- Penetration methods, leaving traces in the form of holes with a diameter of about 3 mm (hardness tests, resistance drilling tests) [16,17];
- Pull-off methods, resulting in irregular breaches having a diameter of about 5 mm [18];
- Methods of small sample collection consisting of cutting out cores or triangular prism-shaped samples, leaving holes or linear dents with a cross-section of up to 10 mm [19,20].

Most commonly, an assessment of the timber structure condition involves selected NDT and SDT methods combined as demonstrated in numerous articles [8,16,21,22].

Non-destructive and semi-destructive tests are currently being strongly developed and are finding application in various branches of industry [23]. When used to test timber constructions, they are a valuable source of information about the structure’s condition. It is important, though, to bear in mind that the results of these tests are affected by such factors as: wood species, wood defects, moisture content, temperature, microbiological factors, and wood age [24]. The influence of particular factors on selected tests and wood species are presented in the articles [25,26,27,28,29,30]. Wood moisture is one of the most significant factors determining its physical and mechanical properties. In turn, moisture poses one of the main threats for heritage constructions, including timber ones [31].

Moisture content (MC) has an influence on the mass of timber, its dimensions and volume, strength, and elasticity properties, and also conductivity and resistance. The moisture of timber is determined by the temperature and humidity of the surrounding air. The substantial influence of moisture on all the physical and mechanical parameters of wood is related to its large internal area constituting groundwork for hygroscopic phenomena [32]. In the wood moisture range from 0% to about 8%, water vapour bonds via monomolecular adsorption; up to a 15% moisture level of wood, polymolecular adsorption phenomena take place; and up to about 22–34%, there are capillary condensation phenomena within sub-microscopic capillaries. This level corresponds to the upper limit of the inner cell wall absorbency, i.e., the fibre saturation point (FSP). At this point, the mechanical properties of wood reach a minimum, whereas swelling becomes maximal [33]. The exact limits of subsequent hygroscopic phenomena are dependent on the wood species, its structure and density, and hence its specific area. In the moisture range between the fibre saturation point and the absolute-dry state, the strength properties of wood increase up to a certain level and then decrease again, except for the compressive strength which reaches a maximum in the state of absolute dryness. Wood exhibits the mechanical properties of the highest values at moisture of about 810% (with the exception of the compressive strength) [34]. A series of studies on the influence of moisture on the physical and mechanical properties were summarized by Gerhards [35]. Currently, the reference timber moisture level for the test results in the case of physical and mechanical properties is assumed to be 12% [36].

This work focuses on issues related to the influence of the moisture content of wood on the results of sclerometric and resistance drilling tests (SDT) for a selection of species of European softwood. As far as building studies are concerned, the most interesting moisture ranges are those up to the fibre saturation level. There are, however, cases where it will be necessary to conduct tests at much higher moisture content levels, even up to complete fibre saturation with water, i.e., members permanently immersed in water, such as wooden piles, palisades, or bridge elements [37,38].

The main aim of the research was to demonstrate the range of influence of moisture on the results of selected NDT and SDT tests, which can be used during in situ tests in buildings. In particular, these relationships are important for the examination of historical and heritage structures. Those structures are usually exposed to increased moisture content and simultaneously require the determination of the actual load-bearing capacity of each structural element. Understanding such relationships provides the opportunity to fully utilize the potential of using NDT and SDT in estimating both physical and mechanical properties of wood in existing structures. Thus, it shall enable the performance of structural analysis and the design of any necessary repairs or reinforcements.

## 2. Materials and Methods

Laboratory experiments were carried out in order to assess the influence of the moisture content of timber elements on the results of non-destructive and semi-destructive tests. The tests were conducted for three species of wood most commonly used in construction in Central Europe: pine, spruce, and fir. Specimens with a cross-section of 175 × 175 mm and a length of 2000 mm were used for extraction of six cuboidal samples with dimensions of 175 × 175 × 320 mm. The samples are a simulation of structural elements, and it was deliberately decided to conduct experiments on a present-day material in order to eliminate the influence of other factors, such as stress condition and age, on the test results. Table 1 presents basic information about the samples.

**Figure 2:**
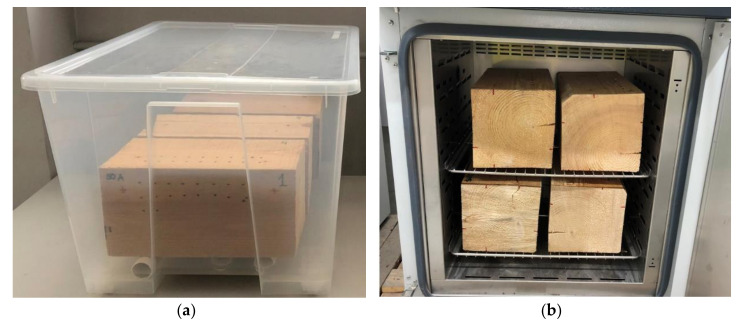
Seasoning of samples: (**a**) moisture-rise chamber; (**b**) wood-drying chamber.

The samples were divided into two groups: the group in which the moisture content was increased and the group in which the moisture content was decreased, starting from the air-dry state. Each group comprised three samples of each species. Individual samples were tested a minimum of three times at different moisture contents. The first group, after a reference test in the air-dry state, was seasoned in climatic chambers at a temperature of 25 °C and air humidity of 95% until the required moisture content level was reached (Figure 2a). Having exceeded the fibre saturation point, a part of each sample was completely immersed in water with an average temperature of 25 °C and was seasoned in this way for the next twelve months. The second group of samples, after the reference test in the air-dry state, underwent a process of gradual drying at a temperature of 45 °C, and next at a temperature of 65 °C in laboratory dryers without forced air convection to an absolute-dry state at a temperature of 100 °C. All the tests on the samples in this group were carried out after cooling in a desiccator to room temperature and after control mass measurements.

**Figure 3:**
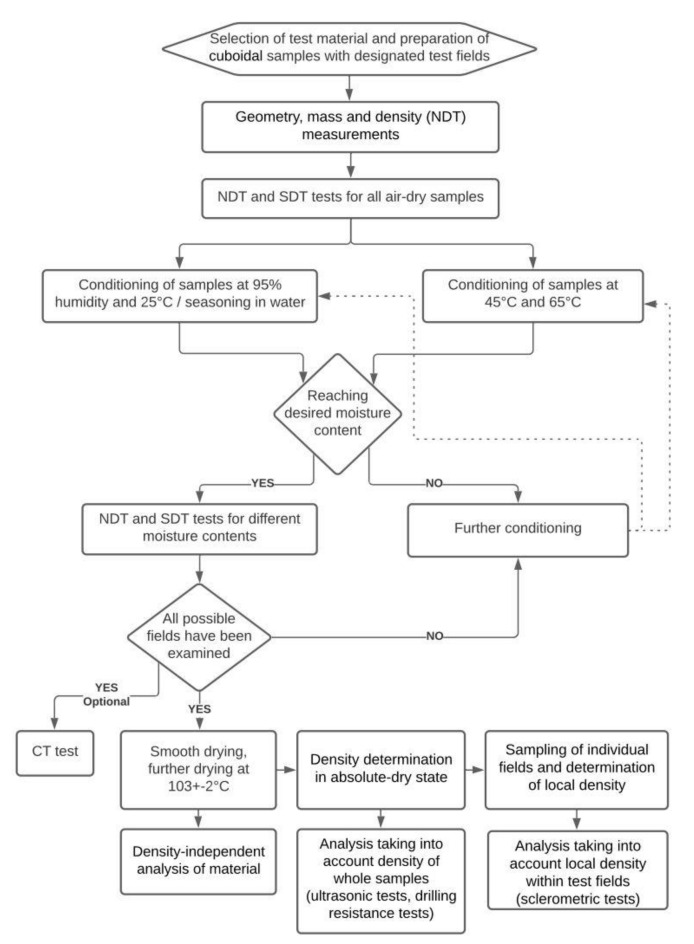
Schematic diagram of research procedure.

**Figure 4:**
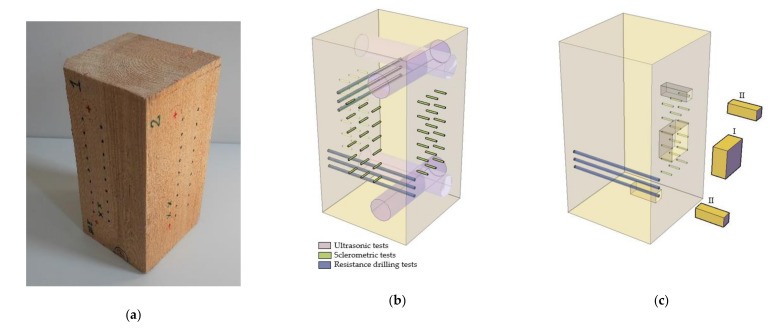
(**a**) Example of a sample; (**b**) Schematic view of test field distribution; (**c**) Illustration of locations of samples for determination of area density in the absolute-dry state (I) and tomography imaging studies (II).

The assessment procedure involved a macroscopic evaluation (identifying places with defects in order to eliminate their influence on the test results), ultrasonic wave propagation tests, sclerometric tests, and resistance drilling tests. Additionally, X-ray computed tomography imaging was performed to visualize the damage structure. All the tests were performed perpendicularly to fibres. During the works, moisture measurements of the elements were conducted using the capacitive method (initial determination if the moisture level reached was close to the desirable one), and after the test cycle, a moisture test was carried out by a means of the oven dry method [36,39]. Next, the density of the samples was found in the absolute-dry state (ρ_0_), and after slitting in the absolute-dry state within every sclerometric test area (ρ_0,s_) from a cuboidal sample with dimensions of 50 × 50 × 25 mm. The test procedure is illustrated schematically in Figure 3. Figure 4 shows an example of a sample with the test areas and points marked and a schematic view of the test area distribution.

**Figure 5:**
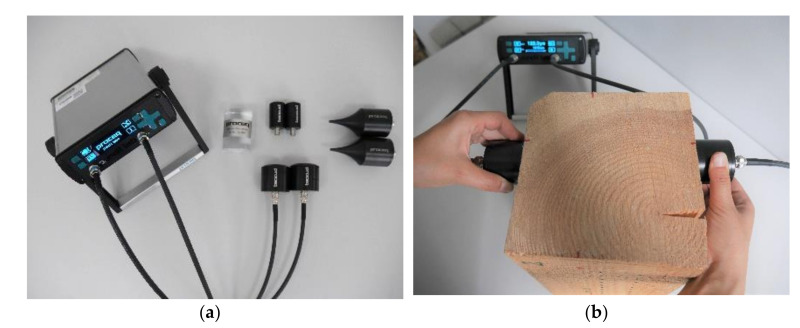
Apparatus for non-destructive test (NDT) studies: (**a**) PunditLab Proceq with accessories; (**b**) Sample under investigation.

Ultrasonic tests were carried out using a PunditLab set produced by Proceq (Schwerzenbach, Switzerland) with transducers operating at a frequency of 54 kHz with an automatic transmitter voltage. Before each use, the device was calibrated on the dedicated calibration rod with an ultrasonic wave passage time of 25.4 μs. The measurements were taken using a coupling agent in the form of a chemically passive polyacrylic gel and both transducers were coated with a thin layer of the gel before each measurement. For each sample, 4 test cross-sections were selected in places found macroscopically to be defect-free (two areas on each side). For each test cross-section of a sample, 3 measurements were taken and the average values were used as the results of the ultrasonic wave propagation velocity between the transducers, i.e., the opposite sides of the sample. The apparatus used in the measurements and an example sample undergoing the test are presented in Figure 5.

**Figure 6:**
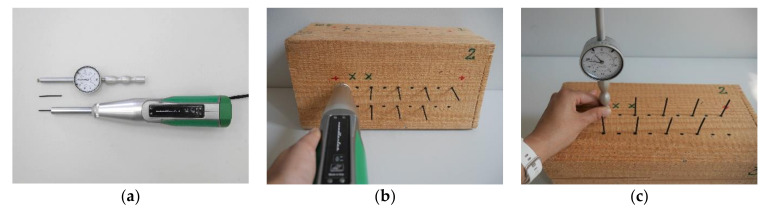
The device used for semi-destructive test (SDT) studies. (**a**) Woodtester Novatest and accessories; (**b**) Dynamic insertion of sclerometer pins; (**c**) Penetration depth readout.

Sclerometric studies were performed using a Woodtester device from Novatest (Ancona, Italy) with an impact energy of 2.4 J, equipped with steel pins having a hardness of 60 HR and a diameter of 2.5 mm with a cone-shaped tip inclined at an angle of 35 degrees and a length of 50 mm, and a clock sensor offering measurement accuracy of 0.01 mm. The device was calibrated on a testing anvil characterized by hardness of >52 HRC. Marking was done on the device in a horizontal position. The quantity measured was the remaining part of the pin that had not penetrated into the material, and the measurement result was taken as the difference between the pin length and quantity measured. The maximum possible pin penetration depth after a fivefold impact, as recommended by the producer, is 40 mm. However, for the species of wood used in the experiment with a high moisture level, three impacts produce a cavity which is greater than the measurement limitation of the device, hence a decision was made to consider the results of a single (PD_1_) and double (PD_2_) hammer impact. After readouts of the first and second impact, the pins were removed using hand tools. For each sample, three test fields were selected on its three sides in places macroscopically evaluated as defect-free. In each field, 18 points were selected for tests under different moisture levels and no fewer than 9 measurements were taken for each area and each moisture level. The device used and an example of a specimen in the middle of testing are shown in Figure 6.

**Figure 7:**
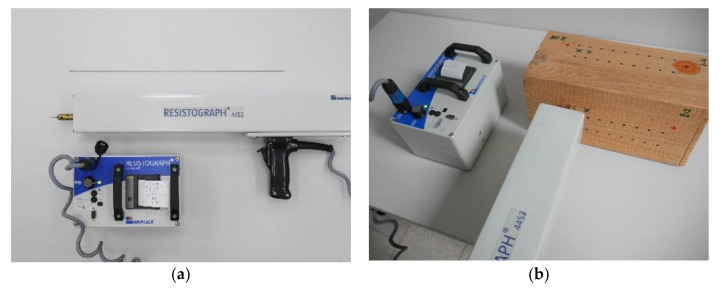
The device used for SDT studies. (**a**) Rinntech resistograph for resistance drilling tests; (**b**) Device conducting a test.

The resistance drilling test was carried out by a means of a 4453-S resistograph device produced by RinnTech (Heidelberg, Germany) with a drilling rate of 40 cm/min and a resolution of 1/100 mm dedicated to tests of wood of average density and equipped with a drill bit with a diameter of 1.5 mm and a head of 3.0 mm. The torque required to keep a constant drilling rate corresponds to wood resistance and is recorded and plotted versus the drill bit penetration depth. The tests were conducted perpendicularly to fibres, away from the defect-occurring zones. The results of the measurements were taken as the quotient of the area under the resistance drilling curve and the drill depth (RM) [40], as a dimensionless quantity dependent on the device used. For each element, 2 reference cross-sections were selected for which resistance drilling tests were performed in the air-dry state, whereas subsequent measurements were taken at various moisture contents in cross-sections situated 15 mm away from the previous one. The apparatus and an example of a specimen in testing are shown in Figure 7.

**Figure 8:**
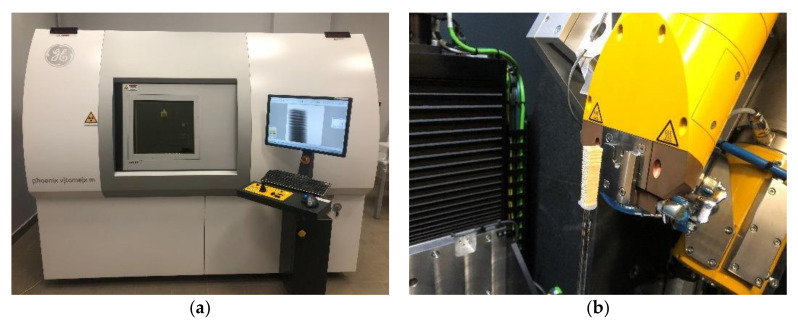
X-Ray CT studies. (**a**) GE Phoenix v-tome-x m device performing a test; (**b**) Sample prepared for a tomography test positioned inside the device.

For a selected pine specimen, X-ray computed tomography was performed in place of the sclerometric and resistance drilling tests to picture the damage to the wood fibre structure. The tests focused on sections with dimensions of 15 × 15 × 40 mm containing holes formed as a result of the SDT carried out at the aforementioned moisture contents MC ≈ 12% and MC ≈ 100%. Tomography of the sections was performed in their air-dry state (MC ≈ 12%) and in the full FSP (MC ≈ 100%) using a GE Phoenix v-tome-x m device (Figure 8a).

The GE Phoenix v-tome-x m device operating on the VG Studio Max (version 3.4) software allows for reconstruction and analysis of the internal element structure based on a series of X-ray images taken while the sample was rotating 360°. Given the different moisture level of the material and slightly different specimen sizes, the parameters of the test were adjusted each time to the element under investigation accordingly. Table 2 lists the basic test parameters and their value ranges used in the experiment.

## 3. Results

**Figure 9:**
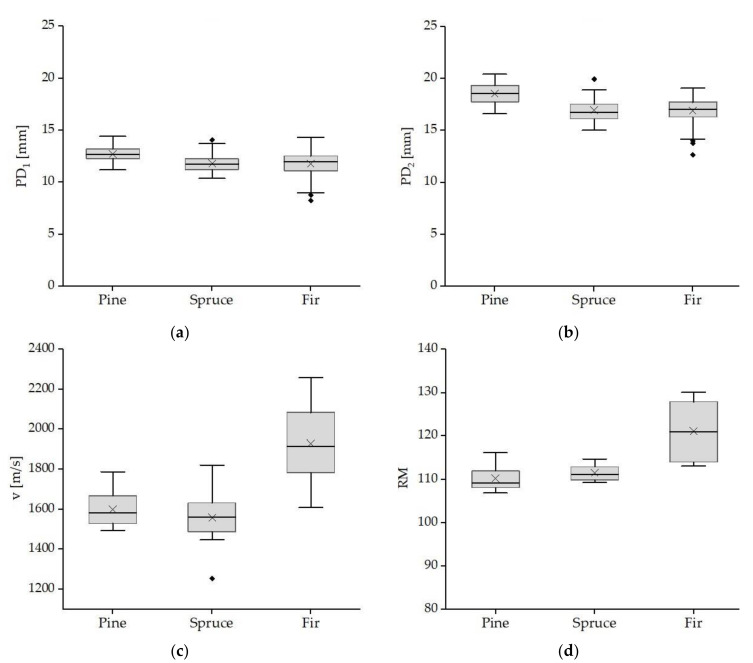
Distribution of test properties in the reference sample for individual wood species. Penetration depth (**a**) after a single impact, (**b**) after a double impact; (**c**) velocity of ultrasonic wave propagation; (**d**) drilling resistance.

First, NDT and SDT tests were carried out on all samples in the air-dry state (moisture in the range 11–13%) as reference tests. The following parameters were evaluated: arithmetic average, median, and standard deviation. Most of the results are characterized by little variance, which indicates high uniformity, with a clear exception in the case of the fir samples. All the results in individual groups, except for the penetration depths PD_1_ and PD_2_ for the fir samples, fit in a normal distribution (according to the Shapiro–Wilk statistical test). The distribution of the characteristics under investigation is presented in Figure 9.

The influence of moisture on the results of the ultrasound, sclerometric, and drilling resistance tests was analysed. The results were divided into a spectrum of hygroscopic moisture—from zero to the fibre saturation point (FSP) approximately equal to MC ≈ 0–30% and capillary moisture (above the fibre saturation point). For moisture above FSP, in order to determine the correlations, the outer points of the hygroscopic range were additionally assumed, for which the moisture content was higher than 25%. Subsequently, various regression models were verified, from which the linear regression method was selected for presentation of results and further considerations. It should be noted that for data above the FSP, the presented coefficients of determination R^2^ were derived from a small amount of data.

Moreover, a possibility was considered to formulate this relation independently of the wood species. Assuming that the physical and mechanical properties of wood are strongly determined by density, the results of the SDT and NDT tests were divided by the density of the sample in the absolute-dry state (ρ_0_). The resultant correlation between the moisture and the quotient of the test results (v, PD_1_, PD_2_, RM) and the density in the absolute-dry state is lower than when each wood species was analysed separately. This confirms that many other factors additionally affect the results of the tests. Therefore, this normalization was omitted in further analyses. In Section 3.1, Section 3.2 and Section 3.3, the results of the tests are summarized.

### 3.1. Ultrasonic Wave Propagation Studies

**Figure 10:**
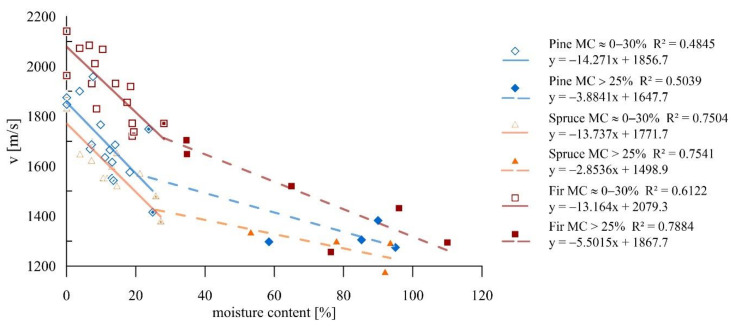
Ultrasonic wave testing for samples of different moisture levels.

The analysis was carried out on the results obtained for the individual species of wood. Generally, there was a decrease in the velocity of the ultrasonic wave passage as the moisture increased. Regardless of the wood species, this drop was more significant in the moisture range below the FSP than above the FSP. A strong correlation was found for spruce and fir timber and a slightly weaker one was for pine timber. The results of the study with defined trend lines and corresponding coefficients of determination are presented in Figure 10.

For all wood species in the moisture range below the FSP, the drop in velocity of the wave passage with a moisture change of one percentage point was 13.7 m/s on average. This represents approximately a 1.0% change in the result with a moisture change of 1% for pine and spruce and 0.8% for the fir. The smallest decrease in the moisture range above FSP was recorded for spruce samples, which was about 2.8 m/s per one percentage point of moisture increase; in this range, the highest decrease was recorded for fir samples and reached 5.5 m/s, which is 0.24% and 0.42% respectively.

### 3.2. Sclerometric Studies

**Figure 11:**
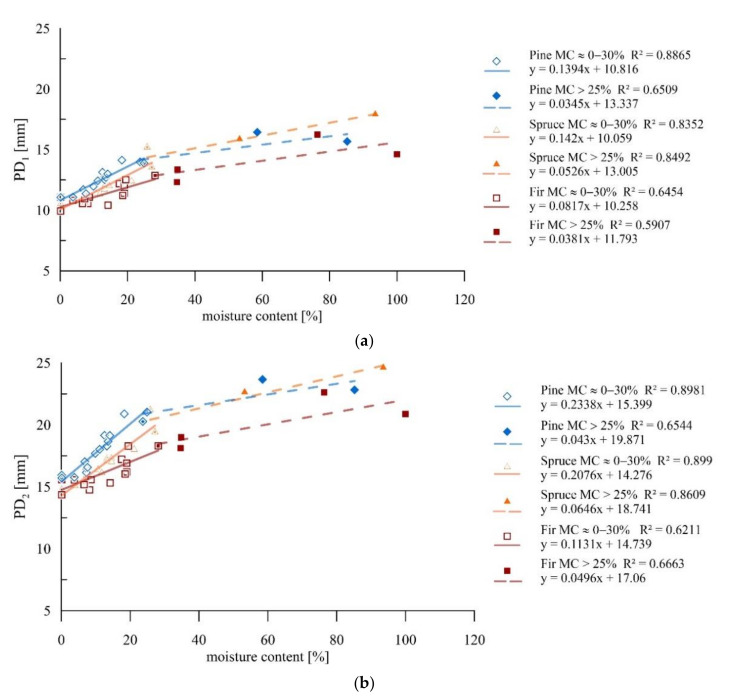
Sclerometric studies on samples of different moisture contents: (**a**) Results for individual wood species for a single impact; (**b**) Results for individual wood species for a double impact.

The sclerometric tests revealed an increase in penetration depth with increasing moisture of the material, which is confirmed by the strong correlations. Analysis was performed of the results obtained for individual wood species for a single and double impact. The results of the tests, accompanied by defined trend lines and corresponding coefficients of determination, are presented in Figure 11a,b. For all the species, there was a strong correlation independently of the number of impacts or moisture content.

Below the FSP, an increase of one percent in humidity caused a reduction in the result (PD_1_ and PD_2_) of approximately 0.9%, with larger modifications occurring for pine and spruce samples than for fir samples. For all wood species after exceeding the fibre saturation point, the influence of moisture on the obtained results decreased and was approximately 0.24% for PD_1_ and 0.22% for PD_2_.

**Figure 12:**
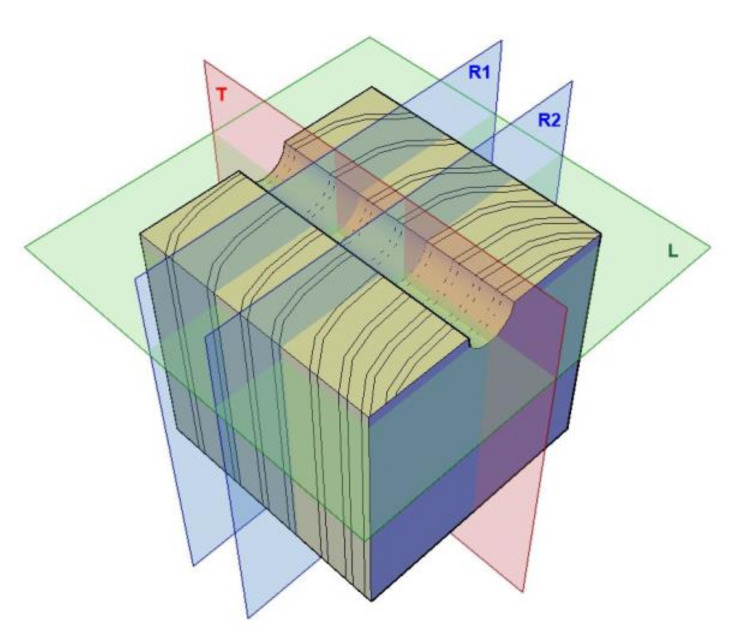
The schematic view of considered sections: R, T, and L.

**Figure 13:**
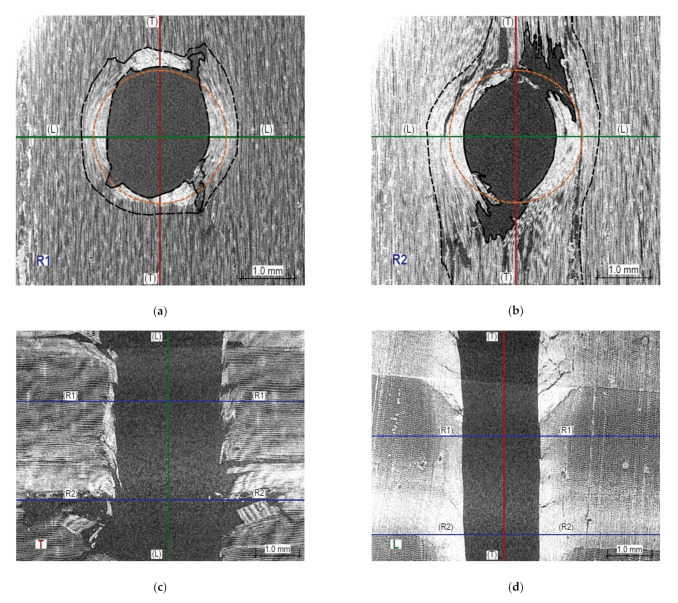
Sections through the opening resulting from the sclerometric test on the sample in the air-dry state: (**a**) Cross-section within earlywood R1—tangential section of wood is visible; (**b**) Cross-section within latewood R2—tangential section of wood is visible; (**c**) Longitudinal section T—radial wood section is visible; (**d**) Longitudinal section L—a cross-section of wood is visible.

**Figure 14:**
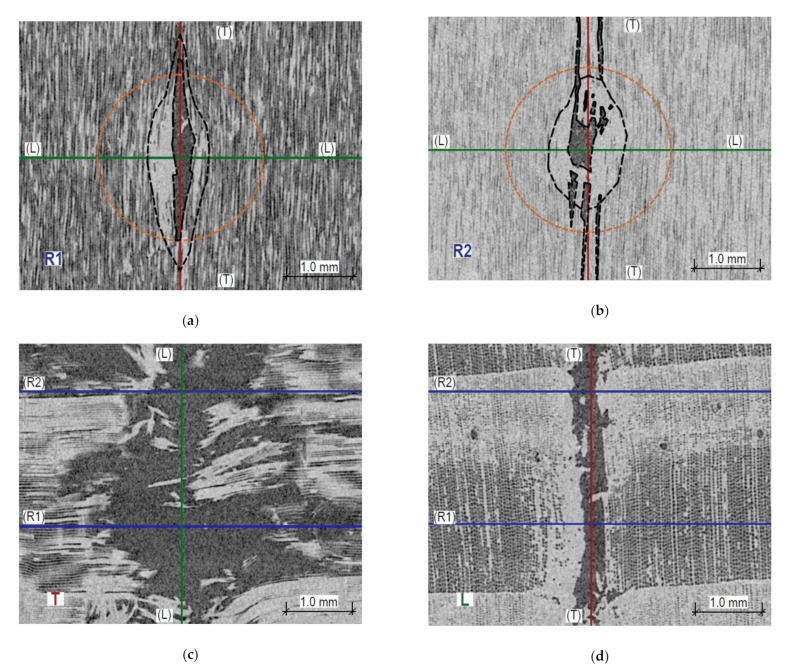
Sections through the opening resulting from the sclerometric test on the sample in the fibre saturation state. (**a**) Cross-section within earlywood R1—tangential section of wood can be seen; (**b**) Cross-section within latewood R2—tangential section of wood can be seen; (**c**) Longitudinal section T—radial section of wood visible; (**d**) Longitudinal section L—cross-section of wood visible.

Figures 13 and 14 present X-ray images of the damage structure in the wood that occurred during the sclerometric test of the samples in the air-dry state and the state of full fibre saturation with water. The naming convention of the sections is given in Figure 12.

It can be seen that the character of the damage to the microstructure is substantially different both in the earlywood and latewood of a given sample as well as for these moisture levels. Penetration of the sclerometer pin through the air-dry wood causes elastic deformation and permanent deformation of individual fibres, most of all in a tangential direction and, to a lesser extent, in a longitudinal direction. The damage is accompanied by a structure integrity breach and brittle fibre fractions, especially in latewood, which manifests through an increase in the damage zone in a longitudinal direction (Figure 13b). The cracks are much larger than the diameter of the sclerometer pin and extend up to 2.8 mm from the hole axis. In Figure 13a,b, the zone of the damaged fibres is outlined with a black dashed line and substantial broadening of this zone is visible in latewood. The test hole has a close to elliptic shape and is smaller than the cross-section of the pin of the sclerometer along the shorter axis by 0.75 mm on average (a cross-section of the sclerometer pin is shown in Figure 13a,b in orange).

In turn, penetration of the sclerometer pin through the saturated wood causes mostly elastic deformation of individual fibres and pronounced cracking along the fibres. Both in earlywood and latewood, the impact hole in a tangential direction is much smaller than the diameter of the sclerometer pin, whereas the fractions in a longitudinal direction are much larger than the pin diameter. In earlywood and latewood, they extend to 1.8 and 3 mm from the opening axis, respectively. In Figure 14a,b, the black dashed line corresponds to the zone of fibres that sustained damage and the area of the compressed fibres is comparable in both cross-sections. The test hole in a tangential direction is considerably smaller than a transverse cross-section of the sclerometer pin (the sclerometer pin cross-section is shown in Figure 14a,b in orange).

### 3.3. Resistance Drilling Studies

**Figure 15:**
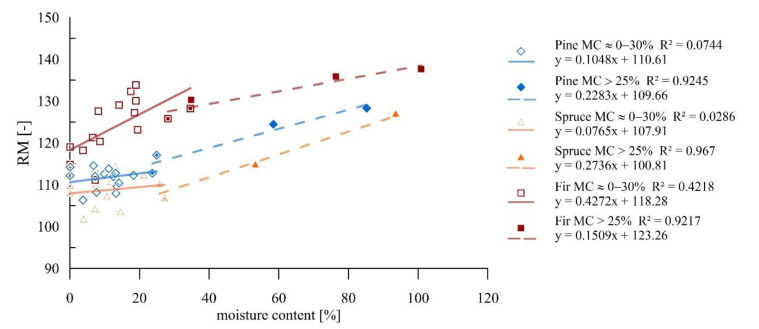
Resistance drilling tests for samples of different moisture contents.

The research indicated a general trend of the development of drilling resistances as the moisture content increases. Nevertheless, the weak correlations in the range below the FSP suggested a weak dependence of drilling resistances on the moisture content in this spectrum, whereas above the FSP, these correlations are very strong. The results of the study with defined trend lines and corresponding coefficients of determination are given in Figure 15.

Below the FSP for fir wood, an increase in moisture content of 1 percentage point contributed to an increase in drilling resistance of about 0.3%. The coefficients of determination obtained in this range for pine and spruce wood did not indicate a significant relationship. In the range above the FSP, an increase in moisture content by 1 percentage point caused an increase in drilling resistance of about 0.2% on average for all wood species.

**Figure 16:**
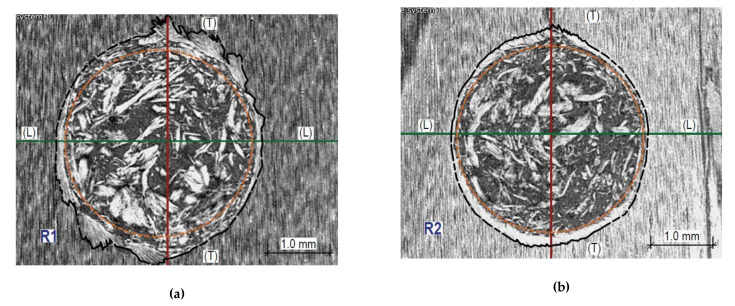

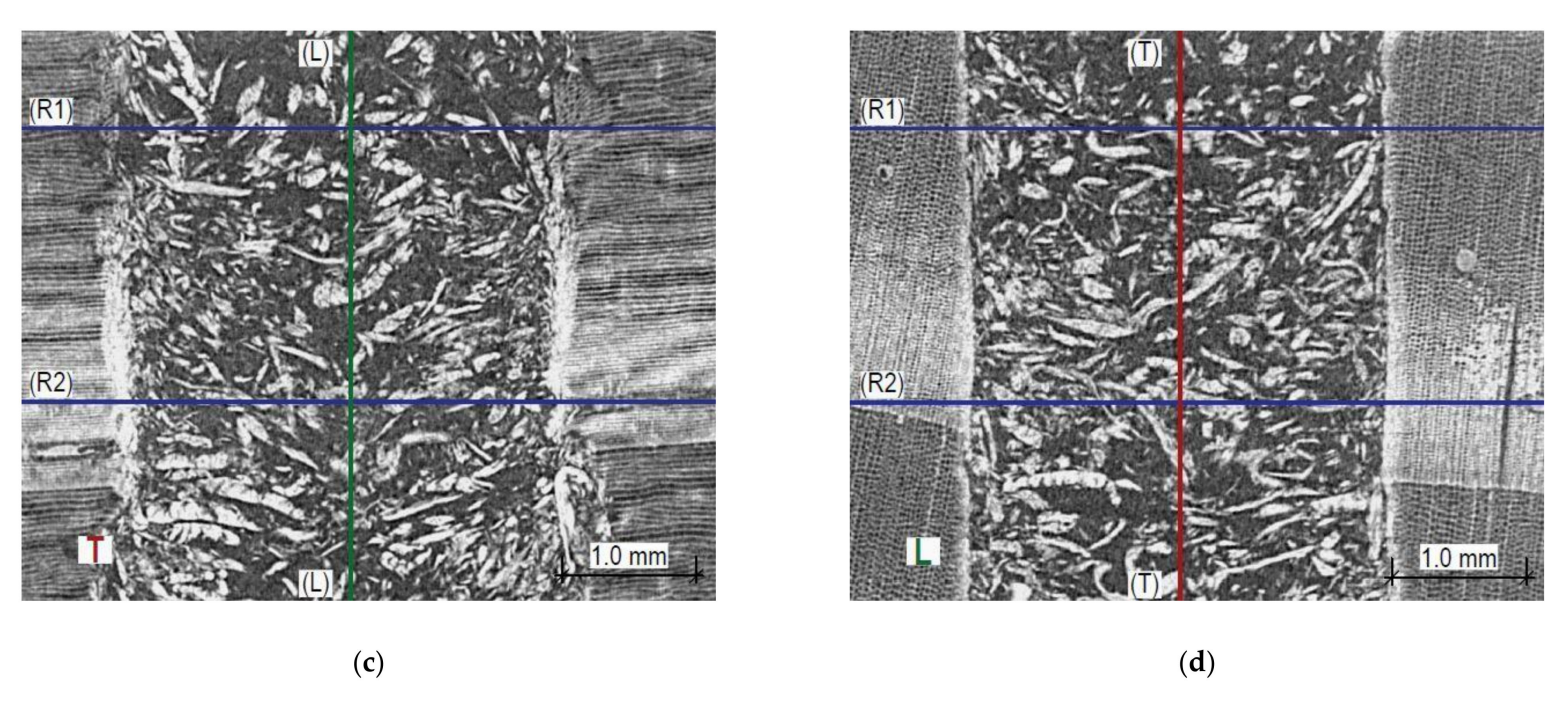
Sections through the opening after the resistance drilling test of the sample in the air-dry state. (**a**) Cross-section within earlywood R1—tangential wood surface visible; (**b**) Cross-section within latewood R2—tangential wood surface visible; (**c**) Longitudinal section T—radial wood surface visible; (**d**) Longitudinal section L—wood cross-section visible.

**Figure 17:**
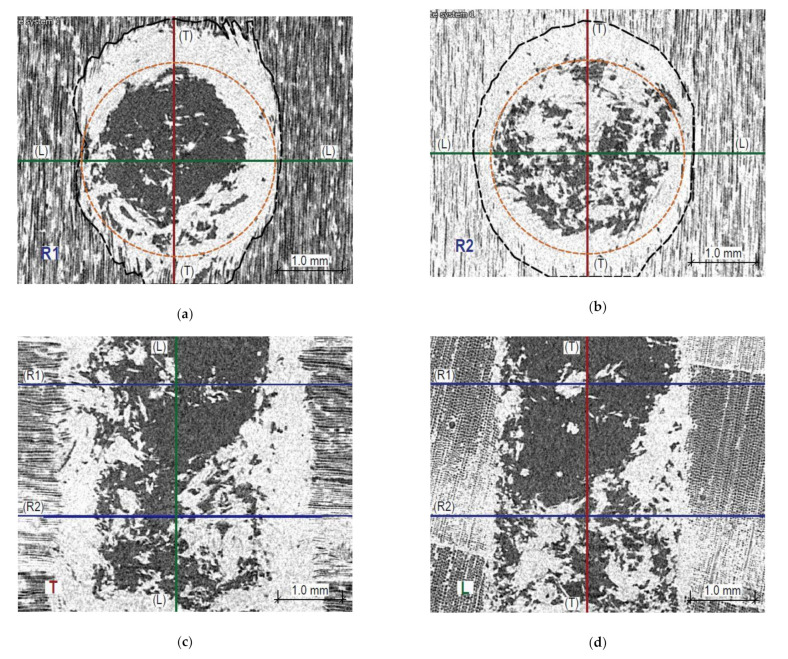
Sections through the opening after the resistance drilling test of the sample in the fibre. (**a**) the tangential wood surface is visible; (**b**) Cross-section within latewood R2—the tangential wood surface is visible; (**c**) Longitudinal section T—the radial wood surface is visible; (**d**) Longitudinal section L—wood cross-section is visible.

In Figure 16 and Figure 17, X-ray images illustrate the wood structure damage that was caused in the resistance drilling tests of the air-dry samples and fully water-saturated samples. The naming convention of the sections is given in Figure 12. In resemblance to the sclerometric tests, it can be seen that the damage character of the microstructure differs both within the earlywood and latewood of a given sample and for different moisture levels.

The damage zone in Figure 16a,b is marked with a black line; the distance between the edge of the damaged fibres, in a direction along the fibres, is on average 4.0 mm in earlywood, whereas in latewood, the distance is 3.5 mm. The test borehole is uniformly filled with fragments of drill cuttings. The zone of the drill operation is shown in Figure 16a,b in orange.

The damage zone is marked with a black line in Figure 16a,b. In a direction longitudinal to the fibres, the damage zone extends to 4.1 mm in earlywood and 3.9 mm in latewood. In Figure 17a,b, the drill operation area is outlined in orange.

## 4. Discussion

The results indicated a relationship between moisture and the data from the NDT and SDT tests. The mechanical properties of the cell wall material strongly differ in the considered ranges (below and above the FSP), which is also reflected in the significance of the impact of moisture content growth on the results of these tests. It was found for all the studies under consideration that these relationships are higher in the range below the FSP than in the range above FSP. Within individual species, a more significant effect was observed for pine and spruce than for fir. One should note that the scatter of results given in the graphs (Figure 10, Figure 11 and Figure 15) could be related to the fact that most of the studies were performed between the R and T directions, which also influenced their scale [29]. Table 3 presents the percentage change of the test results for the increase in moisture by one percentage point for individual methods.

Firstly, ultrasonic wave propagation velocity tests (NDT) were carried out, for which the impact of moisture has already been described in the literature. Among other authors, Sandoz [25] reports a decrease in ultrasonic wave velocity by about 0.8% for each 1% moisture content increase. This value was found only for spruce wood in the 5–30% moisture content range. A broader moisture content range (5–126% for the wood of Douglas fir) was studied by Wang [41], who describe the ultrasonic wave velocity drop by a polynomial function in the whole range of moisture content. The studies were carried out along fibres using a hammer, exciting mechanical waves at one end of the specimen, which imposes substantial limitations for application in existing constructions. However, as the author suggested, it could be functional for timber grading. In the moisture range 12–20%, per 1 percent rise in moisture from the polynomial function, a 1.7% decrease in ultrasonic wave velocity was obtained and for moisture in the range 50–60%, per 1 percent increase in moisture from the polynomial function, a 1.0% drop of ultrasonic wave velocity was noted. In other tests, the ultrasonic wave velocity was reduced by 1.0%; for ultrasound velocity, a correction factor with a decrease in velocity of 0.53% for each 1% increase in moisture content (in the range below 28% moisture content) was proposed for spruce [24].

The results obtained in terms of moisture content below FSP are closest to those obtained by Sandoz [25]. In the range above the FSP, the results were much lower than in the study [41], which first of all may be the result of a different wood species and secondly of the research method used and the polynomial correlation chosen in this study.

Currently, the existing results come from the sclerometric tests carried out by means of a Pilodyn 6J device for Douglas fir wood [42] and various species of pine [43]. The results of these studies suggest that the influence of moisture on the sclerometric test results may depend on the wood species. In this work, a 1.6% decrease for each percentage point of moisture increase was indicated for pine wood. This is almost three times higher in the range below the FSP than the results obtained here for fir wood, and is considerably higher than the results obtained for pine and spruce wood regardless of the number of impacts. This phenomenon may be due to the higher impact energy of the device used in the tests of Llana et al. [43]. There is no data for comparison in the moisture range above the FSP; however, similarly to ultrasound tests, the influence of moisture on the sclerometric results decreased as they increased.

An interesting source of information on the influence of this testing method on the microstructure of wood is the CT scanning. Figure 13 and Figure 14 demonstrate typical images obtained for various samples. There is a significant difference in the nature of microstructure destruction for air-dry samples and in the state of full saturation. The dynamic penetration of the sclerometer needle in the air-dry samples caused destruction in the form of a clear tightening of the lumen of the cells (tracheids) of the earlywood, and in the latewood, it was accompanied by significant cracking (Figure 13d). The tightened cells accumulated on the boundary of the annual growth ring. In a completely different way, the destruction took place in the wood in the state of fibre saturation; there is no brittle cracking between the wood cells (Figure 14d) but only a slight accumulation along the hole left by the sclerometer needle. However, there was a separation in the transverse direction of individual wood fibres over a long distance, as shown in Figure 14a,b. This was most probably caused by water occurring between the micelles, which reduces the cohesion forces between them. This different character of cellular structure destruction below and above the FSP indicated a varying influence of moisture on the sclerometric test results in these ranges. Up to the fibre saturation point, the cohesion forces decrease with increasing moisture content and thus the influence of moisture on sclerometric test results is significant. At the point of fibre saturation, the cohesion forces reach the lowest level; further penetration of free water does not significantly change the mechanical properties of the wood, which is reflected in a reduced effect of moisture on the results of these tests. This further, although much smaller, impact of moisture on the results of the tests could be explained by the decreasing friction forces associated with the presence of increasing amounts of free water. For the dynamic character of this test, the free water contributes to the slip of the smooth surface of the sclerometer needle within the wood fibres with minimal cohesion.

In turn, when it comes to the resistance drilling tests, the literature offers data on moisture impact limited only to living trees [44] thus with moisture levels significantly exceeding those found in building structures. Nevertheless, the study shows a general linear relationship between the increase in drilling resistance with the increase in moisture content (in the MC range of 10–300%) and the possibility of using resistographic methods to estimate the density of woods with different moisture content. It should be noted that the obtained correlations for these tests are lower than for sclerometric and ultrasonic tests; similarly, the magnitude of the moisture impact on the results of the drilling resistance tests is lower. For moisture below the fibre saturation point, in the case of pine and spruce wood, the scatter of results is considerable. Presumably, this is a consequence of the different position of the resistographic test in relation to the entire sample cross-section. This does not allow general conclusions to be drawn, but the analysis of the drilling resistances for individual samples indicated the relationship between the increase in drilling resistances and the increase in moisture even within the moisture content below the fibre saturation point. Table 4 shows the results for selected samples in this moisture spectrum. In the case of fir samples, the obtained correlation curve made it possible to determine the effect of moisture at a change of one percentage point on the below FSP results at 0.3%. In the moisture content range above the fibre saturation point, the change in moisture content results in a change of about 0.2% for pine and spruce samples and 0.11% for fir samples.

Analysis of the tomographic images showed the different types of destruction for ranges above and below the fibre saturation point. The resistograph bit penetration through earlywood in the air-dry sample causes fibre destruction, most of all along the fibres. In this zone, characteristic fibre displacements following the drill’s rotational movement are visible. In latewood, the damage type is similar, but its extent is much lesser. The penetration of the resistograph drill bit through the wood in the state of full saturation causes fibre damage to a much greater extent than in the case of air-dry wood. Characteristic displacements of fibres are visible in a direction consistent with the drill bit rotation manner, but the image is not as clear as for the samples in the air-dry state. Both in earlywood and latewood, there is a compaction of drill cuttings at the borehole walls. In latewood, the damage zone in a tangential and longitudinal direction is comparable. In turn, in earlywood, the greatest damage occurs along the fibres.

The destruction of the wood structure by drilling below the FSP differs from the destruction within the range above the FSP—in particular, the distribution of the remaining drill cuttings in the hole. The drill cuttings for the samples above the FSP range accumulated near the walls of the drill hole and thus additionally increased the resistance to the side of the drill bit in the drilling part.

The drilling resistance test in terms of the wood microstructure is a component of the shearing of individual wood fibres by the drilling tool and the resistance of this material. Both in the moisture range below and above FSP, the drilling conditions between the tool and the material under investigation change with increasing moisture content. In this case, unlike sclerometric tests, the water content increases the friction forces (drilling resistance forces). These forces are superior to the decrease in mechanical properties that occurs in the wood as the moisture content increases in the range below the FSP. Moreover, the decrease in the modulus of elasticity of the wood together with the increase in moisture causes a decrease in the resistance force, which has a significant impact on the drilling process of the material.

Synthesising the results obtained, the linear relationships can be expressed by Equation (1), which allows recalculation of a result of the NDT and SDT tests at a given moisture content MC (X_MC_) to a result expected at moisture of 12% (X_12_). This equation is analogical to the equation used in the literature to recalculate the mechanical properties of wood from a moisture content different from the reference moisture value [45]; a similar relation can also be found in the work [24].

(1) $$X_{12}\;={\;X}_{\mathrm{MC}}\left[1\pm k\left(\mathrm{MC}-12\right)\right]$$

In the equation, for ultrasound tests, a “+” should be used; in turn, for resistographic and sclerometric tests, a “−” should be applied. This corresponds to the positive and negative influence of moisture content on the results of these tests. The k-factor is the percentage change of the test result in the numerical form, determined in Table 3. For example, for ultrasound tests in the range below the FSP for pine wood, the factor k = 0.01 should be applied and for calculations in the range above the FSP, k = 0.003.

For ultrasonic measurements with 54 kHz transducers, a 2.4 J Woodtester sclerometer, and a RinntTech 4453 resistograph for the selected wood species, recalculation of a result to a value expected at a different allowed moisture level (X_i_) can be made using Equation (2) and Table 5, listing the coefficient a, where ΔMC is the difference between the moisture of the element tested and the moisture for which the result is needed.

(2) $$X_{i}\;={\;X}_{\mathrm{MC}}\pm a\cdot \Delta \mathrm{MC}$$

Table 5 presents the average values of the NDT and SDT tests for the 12% reference level and the slope coefficients (a) of the linear relation.

It should be remembered though that these values cannot not be extrapolated beyond the selected wood species and a moisture level of more than 100%. In the case of species of a different anatomic structure or within the same species but with very different densities, additional tests would be required to verify if changes of the results given in the Table 3 and Table 5 are also valid for moisture changes by 1 percentage point. Caution is needed, in particular, in the case of sclerometric tests, for which the literature-reported changes reach even 2% depending on the wood species (tests performed with a different device) [43], and resistance drilling tests, for which there are no publications allowing for a comparison of the results. This is also confirmed by the images obtained through computed tomography of the structural damage to the wood due to the tests. A different damage character for earlywood and latewood of various moisture levels may suggest different results at significantly different shares of these annual growth zones in the element under investigation (which will be reflected in the density of the material).

## 5. Conclusions

For all the tests considered in this work, the influence of wood moisture on the test results cannot be neglected, which is particularly important in the context of studies of existing building objects and the known relations between the changes of the physical and mechanical timber properties and its moisture. It is worth noticing that the moisture content of built-up wooden construction elements is hardly ever equal to 12%—the value for which the correlations are known. Typically, these are individual curves obtained using particular devices and wood species, declared by producers or created by users for their own purposes. Different moisture content leads to complications in interpretation of the results of in situ tests, especially for substantially different moisture levels.

Understanding these relationships provides a reliable assessment of the technical condition of objects and elements of timber construction exposed to extensive moisture, and thus the possibility of efficient repair and preserving considerably more cultural heritage. Depending on the aim of an in situ test and the evaluation of scrutiny level required, it is recommended that Equations (1) or (2) should be used. The authors suggest that recalculations to a different moisture content should be taken into account when the moisture difference is greater than ± 4% with respect to the reference value. Substantial differences in the results are expected, especially in tests of engineering objects constantly exposed to moisture or direct contact with water, such as bridges or piles, for which the moisture content will be high, or facilities that are exposed to weather conditions due to improper operation.

The article describes relationships for the most popular structural timber in Central Europe; it should be noted that these relationships, as well as the general physical and mechanical properties of the wood, are dependent on the habitat conditions, and therefore, the possibility of using the proposed coefficients for other areas is limited. Most importantly, as a completely new feature, the article presents a reference to the influence of moisture on the results of the drilling resistance tests. This device appeared to be the least sensitive to the increase in wood moisture, which may be an advantage of choosing these tests for structures. However, it is worth noting that in contrast to sclerometric tests, a higher result for the drilling resistance is supposed to represent higher mechanical parameters, which in the case of the obtained results may lead to incorrect interpretation of high drilling resistances if the moisture content of the wood is not taken into consideration and, consequently, to overestimation of the load-bearing capacity of the element or object.

## Figures and Tables

**Table 1:** Properties of test samples.

Name	Botanical Name	Nominal Sample Size [mm]	Average Density for 0% Moisture [kg/m^3^]	MC * Reached [%]
Pine	*Pinus sylvestris*	175 × 175 × 320	409.2	0, 4, 7, 10, 12, 14, 18, 25, 58, 85, 90, 95
Spruce	*Picea abies*	175 × 175 × 320	386.7	0, 4, 7, 10, 12, 15, 21, 26, 53, 78, 92, 94
Fir	*Abies alba*	175 × 175 × 320	367.8	0, 4, 7, 10, 12, 14, 19, 28, 35, 65, 76, 96, 102

* The table lists approximate values. The moisture content (MC) values were used in the analysis with an accuracy of 1/100%.

**Table 2:** X-Ray CT test parameters.

Parameter	Value
Source Voltage/Source Current	40–50 kV/90–110 μA
Voxel size	10–12 μm
Filter	none
Exposure time	500 ms
Number of X-rays used to reconstruct a 3D image	3000 pcs

**Table 3:** Summary of percentage changes of NDT and SDT test results for 1% increase in moisture content.

Tests	Pine		Spruce		Fir	
	MC ≈ 0–30%	MC > 25%	MC ≈ 0–30%	MC > 25%	MC ≈ 0–30%	MC > 25%
Velocity of Ultrasonic Pulse Propagation (m/s)	0.998%	0.308%	1.010%	0.235%	0.781%	0.417%
Penetration Depth PD_1_ (mm)	−0.929%	−0.205%	−0.991%	−0.288%	−0.643%	−0.244%
Penetration Depth PD2 (mm)	−1.043%	−0.178%	−1.012%	−0.256%	−0.624%	−0.225%
Drilling Resistance RM (-)	(−0.092%) *	−0.172%	(−0.069%) *	−0.213%	−0.326%	−0.109%

* Results from relationships that did not obtain satisfactory coefficient of determination.

**Table 4:** Summary of the drilling resistance results for selected samples in the moisture range below the fibre saturation point (FSP).

Sample	Mean Resistance Drilling [RM]		
	MC ≈ 4%	MC ≈ 7.5%	MC ≈ 10%
H_1_ Pine Sample	107.3	109.5	113.9
H_2_ Pine Sample	103.2	106.9	111.2
H_1_ Spruce Sample	96.1	99.5	107.1
H_2_ Spruce Sample	94.9	98.3	106.9

**Table 5:** Average values for reference level of MC = 12% and slope coefficient a.

Tests	MC	Pine		Spruce		Fir	
		X_MC_ = 12%	a	X_MC_ = 12%	a	X_MC_ = 12%	a
Velocity of Ultrasonic Pulse Propagation (m/s)	Below FSP	1598.8	**−14.271**	1557.3	**−13.737**	1930.0	**−13.164**
	Above FSP		**−3.884**		**−2.853**		**−5.502**
Penetration Depth PD_1_ (mm)	Below FSP	12.70	**0.1394**	11.81	**0.142**	11.78	**0.0817**
	Above FSP		**0.0345**		**0.0526**	**–**	**0.0381**
Penetration Depth PD2 (mm)	Below FSP	18.55	**0.2338**	16.93	**0.2076**	16.92	**0.1131**
	Above FSP		**0.043**		**0.0646**		**0.0496**
Drilling Resistance RM (–)	Below FSP	110.18	(0.1048) *	111.45	(0.0765) *	121.18	**0.4272**
	Above FSP		**0.2283**		**0.2736**		**0.1509**

* Results from relationships that did not obtain satisfactory coefficient of determination.

## Data Availability

Data is contained within the article.
